# Effect of Photodynamic Antibacterial Chemotherapy Combined with Antibiotics on Gram-Positive and Gram-Negative Bacteria

**DOI:** 10.3390/molecules23123152

**Published:** 2018-11-30

**Authors:** Yana Ilizirov, Andrei Formanovsky, Irina Mikhura, Yossi Paitan, Faina Nakonechny, Marina Nisnevitch

**Affiliations:** 1 Department of Chemical Engineering, Biotechnology and Materials, Ariel University, Ariel 4070000, Israel; yanailizirov1@gmail.com (Y.I.); fainan@ariel.ac.il (F.N.); 2 Shemyakin–Ovchinnikov Institute of Bioorganic Chemistry Russian Academy of Sciences, 117977 Moscow, Russia; formanovsky@yandex.ru (A.F.); ivmikhura@yandex.ru (I.M.); 3 Clinical Microbiology Laboratory, Meir Medical Center, Kfar Saba 4428164, Israel; yossi.paitan@clalit.org.il

**Keywords:** antibiotic resistance, Rose Bengal, PACT, sulfanilamide derivatives

## Abstract

The well-known and rapidly growing phenomenon of bacterial resistance to antibiotics is caused by uncontrolled, excessive and inappropriate use of antibiotics. One of alternatives to antibiotics is Photodynamic Antibacterial Chemotherapy (PACT). In the present study, the effect of PACT using a photosensitizer Rose Bengal alone and in combination with antibiotics including methicillin and derivatives of sulfanilamide synthesized by us was tested against antibiotic-sensitive and antibiotic-resistant clinical isolates of Gram-positive *S. aureus* and Gram-negative *P. aeruginosa*. Antibiotic-sensitive and resistant strains of *P. aeruginosa* were eradicated by Rose Bengal under illumination and by sulfanilamide but were not inhibited by new sulfanilamide derivatives. No increase in sensitivity of *P. aeruginosa* cells to sulfanilamide was observed upon a combination of Rose Bengal and sulfanilamide under illumination. All tested *S. aureus* strains (MSSA and MRSA) were effectively inhibited by PACT. When treated with sub-MIC concentrations of Rose Bengal under illumination, the minimum inhibitory concentrations (MIC) of methicillin decreased significantly for MSSA and MRSA strains. In some cases, antibiotic sensitivity of resistant strains can be restored by combining antibiotics with PACT.

## 1. Introduction

Controlling microbial infections is one of the most serious problems faced by mankind. For decades, antibiotics were considered as the most powerful medications against infections. However, the emergence of bacterial resistance to antibiotics changed this situation dramatically. The appearance of antibiotic-resistant bacterial strains is a global problem, since once they appeared in one region, resistant cells spread rapidly all over the globe [1]. Bacterial strains are considered to be resistant when they are not killed during the course of a standard antibiotic treatment [2]. The main reasons for bacterial resistance lie in misuse, inappropriate use and overuse of antibiotics in humans and animals, including agricultural and veterinary applications that cause antibiotic contamination of the environment [3]. The phenomenon of antibiotic resistance necessitated a search for new alternatives to antibiotic therapy. For example, Herry et al. [4] applied artificial liposomes composed of cholesterol and sphingomyelin to combat Gram-positive pathogens such as *Staphylococcus aureus* and *Streptococcus pneumoniae* which produce pore-forming cytotoxins. These liposomes bind toxins, thus preventing their binding to host cells. The treatment with liposomes was tested in vivo on mice and proven to be effective. The authors propose applying the liposomes either alone or conjugated with antibiotics. Horn et al. [5] suggested using statins and showed that statins in general and simvastatin in particular, protect patients from host cell invasion by a common agent of sepsis, *Staphylococcus aureus*. Simvastatin restricted localization of phosphoinositide 3-kinase (isoform p85) and inhibited actin dynamics needed for bacterial endocytosis. Another approach included repurposing drugs, such as non-steroidal anti-inflammatory agents, for treatment of microbial infections [6]. This approach seems to be time- and manpower-conserving and exhibits some advantages over the development of new drugs for this purpose. In some methods, bacteria were not eradicated but lost their ability to harm the host cells. For example, anti-adhesion therapy proposed by Cozens et al. [7] prevented adherence of bacteria to host cells and tissues and enabled the avoidance of infection, since in many cases adherence is a prerequisite for the infection.

Photodynamic eradication of bacteria, called photodynamic antimicrobial chemotherapy (PACT), holds a special place among the alternatives to antibiotics [8,9,10]. PACT is based on the excitation of a photosensitizer (PS) upon illumination, causing transition of the PS from a low-energy ground state to a high-energy excited state. After excitation, PS molecules participate in either Type I or Type II reactions. The PS either reacts with organic substrates with production of free radicals or radical ions (Type I mechanism) or passes its energy to dissolved molecular oxygen producing reactive oxygen species, ROS (Type II mechanism). In both cases, active products of the reactions interact with bacterial membrane phospholipids and proteins. This causes membrane leakage and leads to cell death [11,12]. PS belong to various chemical groups, such as xanthene and phenothiazine dyes, porphyrin derivatives, phthalocyanines, psoralens, perylenequinonoids and other macrocyclic molecules containing a large number of conjugated double bonds [13]. PACT was found to be effective in the eradication of Gram-positive and Gram-negative bacteria [8,9,13,14,15,16,17,18,19,20,21,22,23]. Use of PS has several advantages over antibiotics: 1. PS can eradicate not only Gram-positive and Gram-negative bacteria but also protozoa, viruses and fungi, whereas antibiotics often have a very narrow spectrum of antibacterial activity; 2. Contrary to antibiotics, the use of PS does not induce resistance in bacteria; 3. The probability of causing mutagenic effects upon PS application is very low; 4. PS were found to be active not only against planktonic antibiotic-sensitive bacteria but also against resistant strains and biofilm-forming bacterial species [24].

Another approach to bacterial inhibition is based on a powerful idea of combining antimicrobials with different cytotoxic mechanisms. Konaté et al. [25] tested the antimicrobial action of alkaloid compounds from *Cienfuegosia digitata* Cav. (Malvaceae) in combination with methicillin and ampicillin. This combined treatment exhibited a synergistic inhibitory effect against microorganisms resistant to these antibiotics (MRSA/ARSA). Several works reported use of antibiotics combined with PS. In some cases, this combination exhibited a synergistic effect and showed a principle possibility of recovering the bacterial cells’ sensitivity to antibiotics [26,27,28,29,30].

In the present work, the sensitivity of resistant hospital isolates of *Staphylococcus aureus* and *Pseudomonas aeruginosa* to the photosensitizer Rose Bengal alone and in combination with other antimicrobial agents was studied.

## 2. Results

### 2.1. Characterization of Sulfanilamide Derivatives

Yields, melting points and NMR data for the synthesized sulfanilamide derivatives (**II*a***–***n***) and their precursors (**I*a***–***n***) are given in Appendix A.

### 2.2. Characterization of Hospital Isolates of P. aeruginosa and S. aureus

Two different bacterial species, one Gram-negative and one Gram-positive were used in the study. *P. aeruginosa* and *S. aureus* were chosen since both species represent major human pathogens which are often associated with multi-drug resistant strains, difficult to treat. Bacteria *S. aureus* belong to the most common human Gram-positive commensal pathogens and are generally divided into two groups according to their resistance to the antibiotic methicillin: methicillin-resistant *S. aureus* (MRSA) and methicillin-sensitive *S. aureus* (MSSA). Both groups are related in our study. *P. aeruginosa* is a common Gram-negative opportunistic human pathogen of considerable medical importance. In general, *P. aeruginosa* isolates are divided according to their antibiotic resistant patterns to sensitive or multi-drug resistant isolates, often according to their resistance to ceftriaxone and other 3rd generation cephalosporins. We therefore decided to study both ceftriaxone sensitive and multi-drug ceftriaxone resistant *P. aeruginosa* isolates.

Bacterial strains used in this study were obtained from different clinical or screening samples. Isolates of *P. aeruginosa* and *S. aureus* were obtained from hospital in-patients hospitalized due to different infections or from MRSA screening samples in the case of MRSA. The isolated strains were tested for sensitivity to different antibiotics as described in the Materials and Methods section. Five isolates of *P. aeruginosa* strains (strains 68147, 68161, 68166, 68172 and 64606) were sensitive to ceftriaxone and to most other antibiotics and five other isolates (strains 63917, 63982, 62817, 610656 and 611908) were ceftriaxone resistant and exhibited multidrug resistance to 13 to 15 antibiotics. One MSSA isolate (strain 611971) was sensitive to the tested antibiotics and 5 other MRSA isolates (strains 69654, 69740, 69430, 69621 and 69397) were resistant to 2 or 4 antibiotics. Two ATCC strains (ATCC 25668 *P. aeruginosa* and ATCC 11541 MSSA) were used as control strains.

### 2.3. PACT against MSSA and MRSA Strains

*S. aureus* cells were examined for their sensitivity to Rose Bengal under exposure to white light and minimum inhibitory concentration (MIC) values of Rose Bengal were determined for sensitive and antibiotic-resistant strains (Table 1). The tested strains exhibited sensitivity to the PACT treatment. However, the MIC of Rose Bengal under illumination was the lowest for the control MSSA (0.625 mg/L), whereas the MIC values for the hospital isolates ranged from 1.25 to 2.5 mg/L. In the dark controls, MIC of Rose Bengal was 20 mg/L for both MSSA and MRSA strains.

### 2.4. PACT Combined with Methicillin against MSSA and MRSA

The MIC of methicillin was determined for MSSA and MRSA strains in the absence and in the presence of Rose Bengal, in order to examine whether a combination of photodynamic and antibiotic treatments with different antibacterial mechanisms can increase the sensitivity of bacterial strains to antibiotics. The combined experiments were carried out in the presence of sub-MIC concentrations of Rose Bengal, chosen as half of the MIC value for each strain, in order to exclude any masking effect of Rose Bengal. The results presented in Table 1 show that MIC values of methicillin were 8 to more than 16-fold higher for the MRSA strains than for the MSSA strains. Upon application of sub-MIC concentrations of Rose Bengal to both MSSA and MRSA cells, the MIC of methicillin dropped very drastically, whereas in control experiments Rose Bengal alone at the same concentrations caused no inhibition.

**Figure 1:**
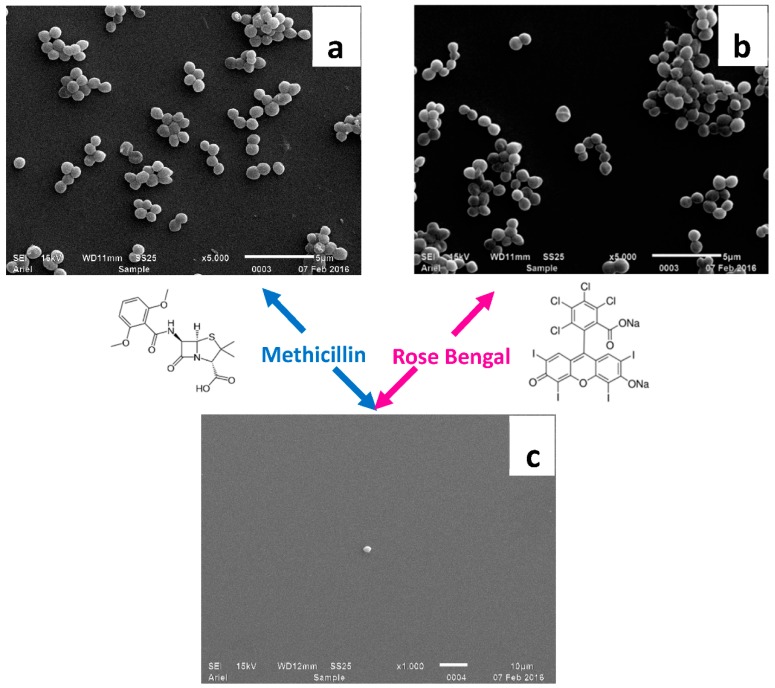
SEM micrographs of MRSA 69397 cells in the presence of 9.5 mg/L of methicillin (**a**), 0.625 mg/L of Rose Bengal (**b**) and 9.1 μg/L of methicillin combined with 0.625 mg/L of Rose Bengal (**c**).

Since in the case of MRSA strains the MIC of methicillin decreased very significantly, even below the initial MIC value for MSSA, it may be concluded that it is possible to restore the MRSA cells’ sensitivity to methicillin. MRSA 69397 cells, which showed a very high response to the combined treatment, were examined by the SEM technique. For this purpose, the cells were treated only by methicillin at the sub-MIC concentration, only by PACT at the sub-MIC concentration of Rose Bengal and by the combined antibiotic-PACT treatment at sub-MIC concentrations of both Rose Bengal and methicillin, when the latter was determined in the presence of sub-MIC concentration of Rose Bengal (Table 1). In the two first cases, the cells remained intact and were unaffected by each of the factors separately (Figure 1a,b). However, when they were treated at sub-MIC concentrations of Rose Bengal and by methicillin at very low concentration (9.1 μg/L), the bacteria were inhibited and live cells were hardly found (Figure 1c).

**Figure 2:**
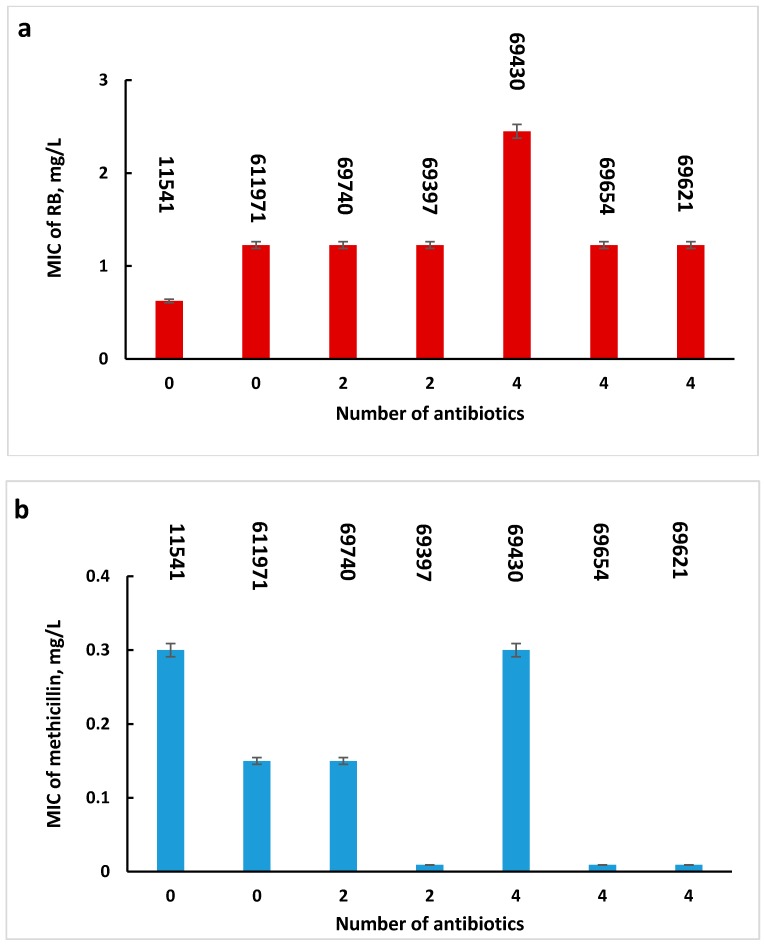
MIC values of Rose Bengal (**a**) and of methicillin in the presence of sub-MIC concentrations of Rose Bengal (**b**) for MSSA and MRSA strains resistant up to 4 antibiotics. Error bars represent standard errors.

In order to determine whether the sensitivity of MRSA cells to PACT and to the combined antibiotic-PACT treatment depended on the number of antibiotics to which these cells were resistant, MIC values of Rose Bengal alone and of methicillin in the presence of sub-MIC concentrations of Rose Bengal were presented against the number of antibiotics (Figure 2a,b, respectively). Figure 2 shows that in both cases, the sensitivity was found to be independent of the number of antibiotics to which the bacteria are resistant (R^2^ = 0.3437, Figure 2a and R^2^ = 0.1405, Figure 2b). Moreover, the type of antibiotics to which MRSA strains are resistant does not affect the restorability of cell sensitivity to methicillin.

### 2.5. Antibacterial Activity of Sulfanilamide Derivatives Alone and Combined with Rose Bengal

**Figure 3:**
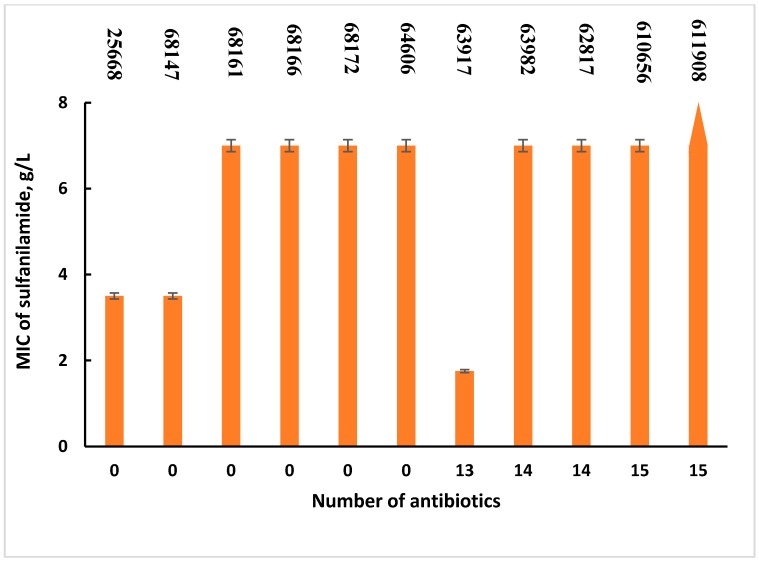
MIC values of sulfanilamide for *P. aeruginosa* strains resistant up to 15 antibiotics. Error bars represent standard errors.

The antibacterial activity of sulfanilamide and its derivatives was tested against hospital isolates of *P. aeruginosa*. First, the antibiotic-sensitive and resistant strains were tested for sensitivity to sulfanilamide alone. Figure 3 shows that MIC values vary for both sensitive and resistant strains. As can be seen from Figure 3, the MIC values of sulfanilamide were 3.5 g/L or less for only two sensitive and one resistant strains, whereas the rest of the cells had a MIC of 7 g/L or more, which is above the solubility of sulfanilamide in the aqueous phase.

It was interesting to evaluate whether the sensitivity of the cells to sulfanilamide depended on their resistance to other antibiotics and whether the MIC values of sulfanilamide were related to the number of antibiotics to which the cells were resistant (Figure 3). In addition, the correlation coefficient was shown to be very low (R^2^ = 0.055). It can therefore be concluded that there is no correlation between the cells’ sensitivity to sulfanilamide and to other antibiotics.

Because of the poor response of most of the *P. aeruginosa* cells to sulfanilamide, the sensitivity of cells to Rose Bengal alone and in combination with sulfanilamide was studied for only two strains—the sensitive 25668 strain and the resistant 63917 strain, which showed low MIC values of sulfanilamide.

Table 2 shows that both strains were inhibited by PACT and that the MIC of Rose Bengal was lower for the sensitive strain 25668. When treated with sulfanilamide combined with PACT in the presence of sub-MIC concentrations of Rose Bengal, the MIC values of sulfanilamide remained unchanged. Contrary to the combined antibiotic-PACT treatment, a combination of sulfanilamide and PACT did not exhibit any decrease in the MIC of sulfanilamide.

The effect of sulfanilamide and its new derivatives **II*a***–***n*** was studied only for the resistant 63917 strain, which was chosen due to its highest sensitivity for sulfanilamide. The experiments for determination of MIC values of sulfanilamide and compounds **II*a***–***n*** were performed with their saturated solutions in the initial test tube. For sulfanilamide it was 7 g/L, which corresponded to its solubility [31]. The experiments showed that none of the **II*a***–***n*** compounds inhibited the *P. aeruginosa* cells and in all cases the MIC values were higher than the solubility of these compounds in aqueous solutions (Table 3).

## 3. Discussion

Two approaches were studied in the present work in an attempt to promote methods for the treatment of antibiotic-resistant strains: PACT and a combination of PACT with methicillin in the case of MRSA strains and with sulfanilamide in the case of multi-resistant *P. aeruginosa*. As demonstrated here, multidrug resistant strains of *S. aureus* can be successfully inhibited by PACT, where the MIC values of Rose Bengal for resistant strains were of the same order of magnitude as for sensitive strains. Sensitive and resistant strains of *P. aeruginosa* however were inhibited at much higher concentrations of Rose Bengal when compared to *S. aureus*. The efficiency of PACT against resistant bacteria was a good reason for a trial to restore their sensitivity to antibiotics by combining them with a PS.

MRSA cells differ from MSSA cells on the genetic level. MRSA cells bear an additional gene, mecA, in their chromosome, which encodes for a penicillin-binding protein PBP-2a that has low affinity for methicillin and remains inactivated upon exposure of the cell to the antibiotic, contrary to four other PBPs (PBP1-PBP4) normally present in *S. aureus* cells [32,33,34]. The normal PBPs catalyze crosslinking reactions of transpeptidation which occur on the external surface of the cytoplasmic membrane. In MSSA cells, methicillin binds to the transpeptidation region of normal PBPs and, as a consequence, causes inhibition of the transpeptidation reaction. The lack of crosslinking of the peptidoglycan leads to mechanical weakening of the cell envelope, release of the cytoplasmic content and cell death. In MRSA cells, after inhibition of normal PBP proteins by methicillin, PBP-2a maintains its activity and takes over the functions of PBP, ensuring crosslinking of the glycan chains in the peptidoglycan. Although PBP-2a does not completely compensate for the normal functioning of the PBP1-PBP4 and the cells growing in the presence of methicillin have reduced crosslinking of the peptidoglycan, this limited rate of crosslinking is sufficient for providing not only survival of the cells but also their propagation [32,33]. Since PACT is known to affect first of all components of the bacterial membrane, a combination of methicillin and PACT seemed to us to be a powerful idea, where the role of methicillin was to inhibit normal cell PBP1-PBP4 and the role of the PS was to interfere with the functioning of PBP-2a by destroying the weakened cell peptidoglycan. Indeed, the results of the present study show that treatment of the MRSA cells by methicillin combined with Rose Bengal at sub-MIC concentrations led to a recovery of the cells’ sensitivity to methicillin, where in most cases the MIC values of methicillin dropped drastically, even below the MIC level of MSSA cells. The observed phenomenon probably exhibited a synergistic character, thus enabling a reduction in applied doses of both drugs and preventing the appearance of resistant cells.

Other successful attempts to combine antibiotics and PACT have been reported recently by others and by us [26,27,28,29,30,35]. Barra et al. [36] showed the combined action of PS and antibiotics against biofilms of clinical isolates of gentamicin-resistant Gram-positive bacteria of the *Staphylococcus* genus: *S. aureus*, *S. epidermidis* and *S. hemolyticus*. Gentamicin was applied in combination with 5-aminolevulinic acid (5-ALA) which does not exhibit photodynamic activity but acts as a metabolic precursor for the endogenous PS protoporphyrin IX (PpIX). Pre-treatment of Gram-positive bacteria biofilms with 5-ALA under illumination was found to make them more sensitive to gentamicin. The authors explain the observed phenomenon by the profound damage to membranes, protein breakdowns and crosslinking, sugar modifications, peroxidation of lipids and harm to the sugars and bases of ribonucleic acids caused by the photodynamic treatment. Due to these effects, penetration of gentamicin through the bacterial membranes was significantly improved. This enabled the antibiotic to bind the 30S subunit of the bacterial ribosome, thus depressing protein synthesis and causing cell death [35].

Efficient eradication of *S. aureus* biofilms was achieved after treating cells first with meso-tetra (*N*-methyl-4-pyridyl) porphine tetra tosylate under illumination and then with vancomycin [27]. Amplification of antibiotic activity by PACT was shown for *S. aureus* and *P. aeruginosa* cells treated with methylene blue or meso-tetra(*N*-methyl-4-pyridyl)porphine tetra tosylate at sub-lethal concentrations [36]. Similar results were achieved when planktonic and biofilm cultures of *Burkholderia cepacian* were exposed to PS combined with tobramycin, ceftazidime, chloramphenicol or ciprofloxacin [26].

Several attempts were made to conjugate antibiotics with PS and these conjugate molecules were shown to have amplified antibacterial activity compared to each component separately. Xing et al. [37] synthesized a porphyrin-vancomycin conjugate, which was found promising for inactivation of not only vancomycin-sensitive but also vancomycin-resistant enterococcal strains. Enhanced photodynamic inactivation of bacteria was attributed to effective adhesion of vancomycin to bacterial envelopes, so that vancomycin was used as an affinity ligand. Cahan et al. [38] demonstrated that Rose Bengal linked covalently to penicillanic acid or kanamycin was effective against pathogenic *S. aureus* and *E. coli* bacteria. The achieved effect enabled the authors to propose that the use of antibiotic-PS conjugates may not only help solve the problem of bacterial drug resistance but may also reduce side effects of antibiotics in patients. Combined antibiotic-PACT therapy has good prospects for wide applications in the treatment of local and systemic external infections. For internal infections, photodynamic excitation of PS should be replaced by alternative methods, for instance chemiluminescent or sonodynamic activation [17,23].

The antimicrobial activity of compounds from the sulfanilamide group is based on competitive inhibition of the enzyme dihydropteroate synthase, which catalyzes conversion of a para-aminobenzoic acid into folic acid necessary for cell replication [39]. Although the inhibitory mechanism of the sulfanilamide compounds is totally different from that of PACT, a combination of sulfanilamide and Rose Bengal did not yield any increase in the sensitivity of antibiotic-sensitive and antibiotic-resistant *P. aeruginosa* cells to sulfanilamide. Contrary to the effect of the combined application of methicillin and Rose Bengal when these two factors were targeted against the cytoplasmic membrane of *S. aureus*, the combined application of sulfanilamide and Rose Bengal against *P. aeruginosa* was not effective. The absence of any additional effect of a combination between sulfanilamide and PACT may be explained by very low rates of bacterial envelope damage by Rose Bengal at sub-MIC concentrations, which was not sufficient for enhancing the action of sulfanilamide. It is probably preferable to choose antimicrobials which affect the same cell sites in order to achieve amplification of their activity.

## 4. Materials and Methods

### 4.1. Materials and Bacterial Strains

Rose Bengal, sulfanilamide, reagents for synthesis and solvents were purchased from Sigma-Aldrich Chemie GmbH (Schnelldorf, Germany). Methicillin was purchased from TOKU-E (Bellingham, WA, USA), growth media Brain Heart broth (BH) and Brain Heart agar (BHA) were purchased from Merck (Darmstadt, Germany) and Antibiotic medium 3 for minimal inhibitory concentration (MIC) studies was purchased from Becton Dickinson & Company (Le Pont de Claix, France). Antibiotic discs were purchased from Abtek Biologicals Ltd. (Liverpool, UK). Antibiotic sensitive and resistant strains of *S. aureus* and *P. aeruginosa* were hospital isolates from the Meir Medical Center (Kfar Saba, Israel). *S. aureus* strain ATCC 11541 and *P. aeruginosa* ATCC 25668 strains were purchased from ATCC (Manassas, VA, USA).

### 4.2. Synthesis of Sulfanilamide Derivatives

**Figure 4:**
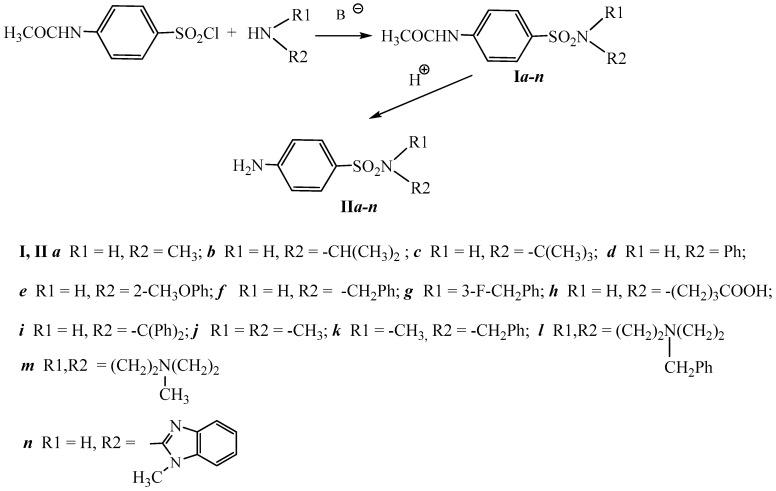
Scheme of the synthesis of sulfanilamide derivatives (**II*a***–***n***).

New sulfanilamide derivatives **II*a***–***n*** were synthetized according to the scheme presented in Figure 4.

Details of the synthesis of compounds from series I and II are specified below.

#### 4.2.1. *N*-Methyl-4-acetylamino Benzenesulfonyl Amide (**I*a***)

5.4 g (80 mmol, 2.0 equiv.) CH_3_NH_2_·HCl were added to a solution of 8.48 g (80 mmol, 2.0 equiv.) Na_2_CO_3_ in 85 mL of H_2_O at 0 °C. Then, 9.34 g (40 mmol, 1 equiv.) 4-acetylamino benzenesulfonyl chloride were added in small portions (0.5 g each) for 1 h under stirring. The reaction mixture was stirred for 1.5 h at room temperature and 0.5 h at 80 °C. After the end of the reaction, the reaction mixture was adjusted to pH 6 with 1 M HCl solution. The precipitate was separated, washed with H_2_O (500 mL) and dried in vacuum under KOH.

#### 4.2.2. General Procedure for Synthesis of Compounds (**I*b***–***n***)

3.24 mL (23 mmol, 0.77 equiv.) of Et_3_N was added to 30 mmol (1 equiv.) of substituted amine in 30 mL of dry DMF. 32 mmol (1.1 equiv.) of 4-acetylamino benzenesulfonyl chloride were then added in small portions (5 portions for 1 h) under stirring and cooling in a water bath (10–15 °C). The reaction mixture was stirred for 12 h at room temperature. After the end of the reaction, the reaction mixture was poured into 100 mL of 1 M HCl and 50 mL of a saturated NaCl solution were added under cooling (10 °C). The precipitate was separated, washed with 200 mL H_2_O and dried in vacuum under KOH.

#### 4.2.3. Sulfanilic Acid *N*-Methyl Amide (**II*a***)

12 mL of concentrated HCl were added to a suspension of 5.50 g (24.12 mmol) **I*a*** in 40 mL of MeOH and were refluxed for 1 h in a flask equipped with a reflux condenser. After the end of the reaction, the reaction mixture was poured into 250 mL of 10% NaHCO_3_ in H_2_O. The precipitate was separated, washed with 200 mL H_2_O and dried in vacuum under KOH.

#### 4.2.4. General Procedure for Synthesis of Compounds (**II*b****–**n***)

10 mL of concentrated HCl were added to a suspension of 25 mmol *N*-substituted 4-acetylamino benzenesulfonyl amide in 40 mL of MeOH and the reaction mixture was refluxed for 1 h in a flask equipped with a reflux condenser. After the end of the reaction, the reaction mixture was poured into 150 mL of 10% NaHCO_3_ in H_2_O. The precipitate was separated, washed with 200 mL H_2_O and dried in vacuum under KOH.

### 4.3. Characterization of the Synthesized Sulfanilamides

**Figure A1:**
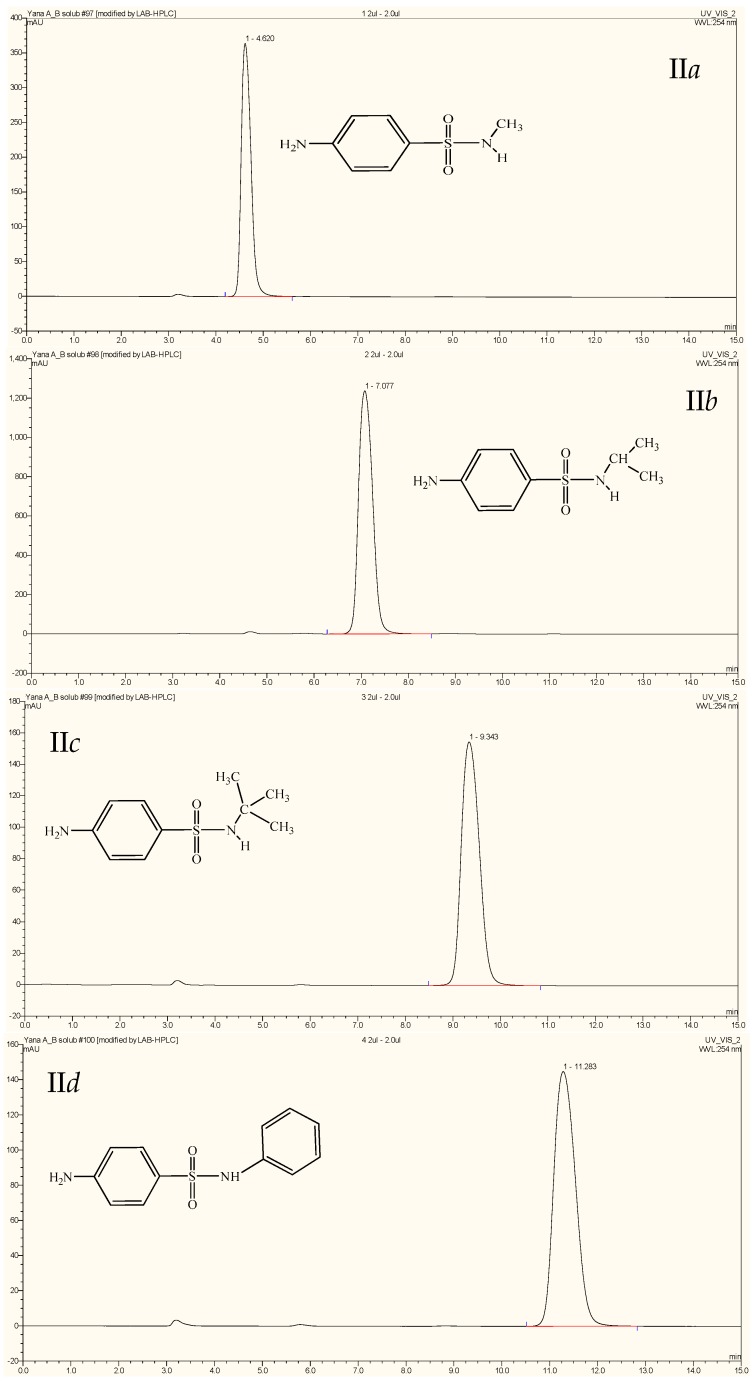

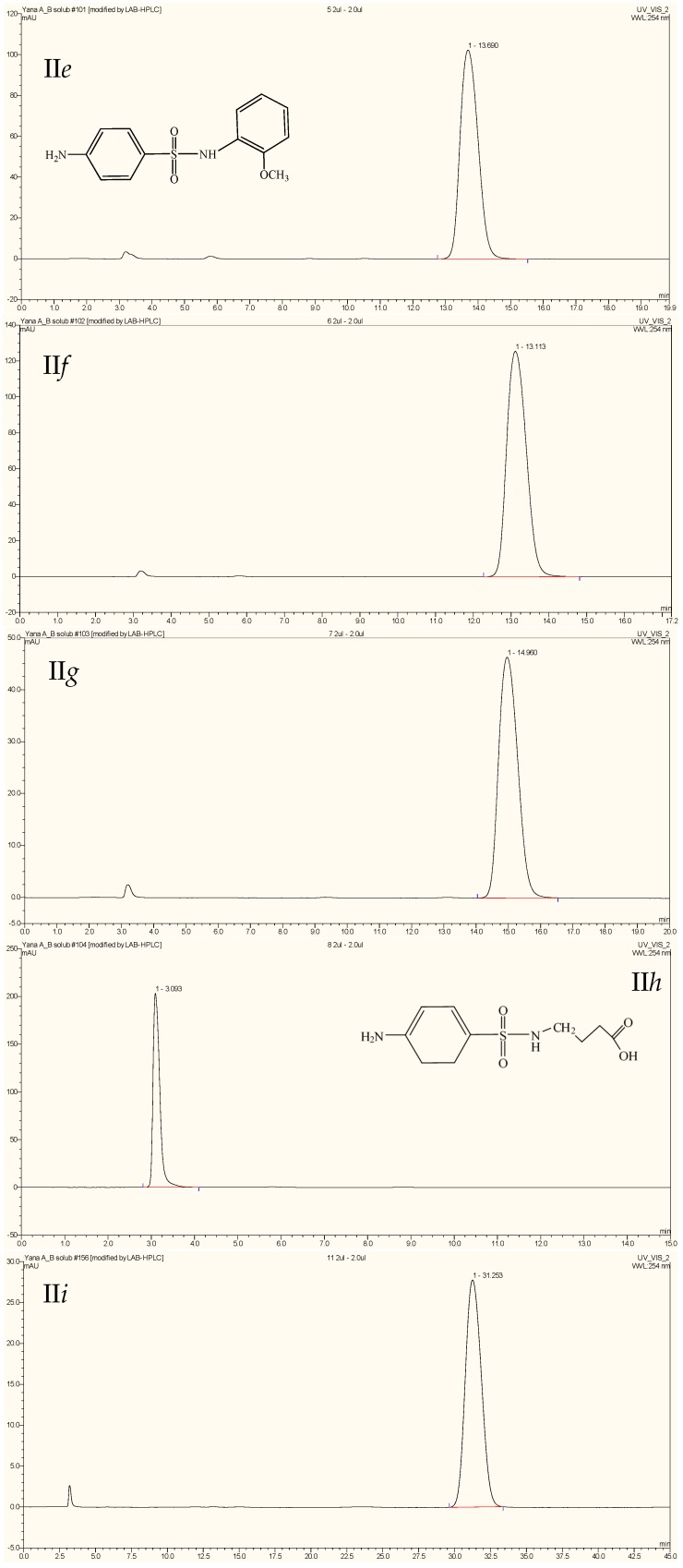

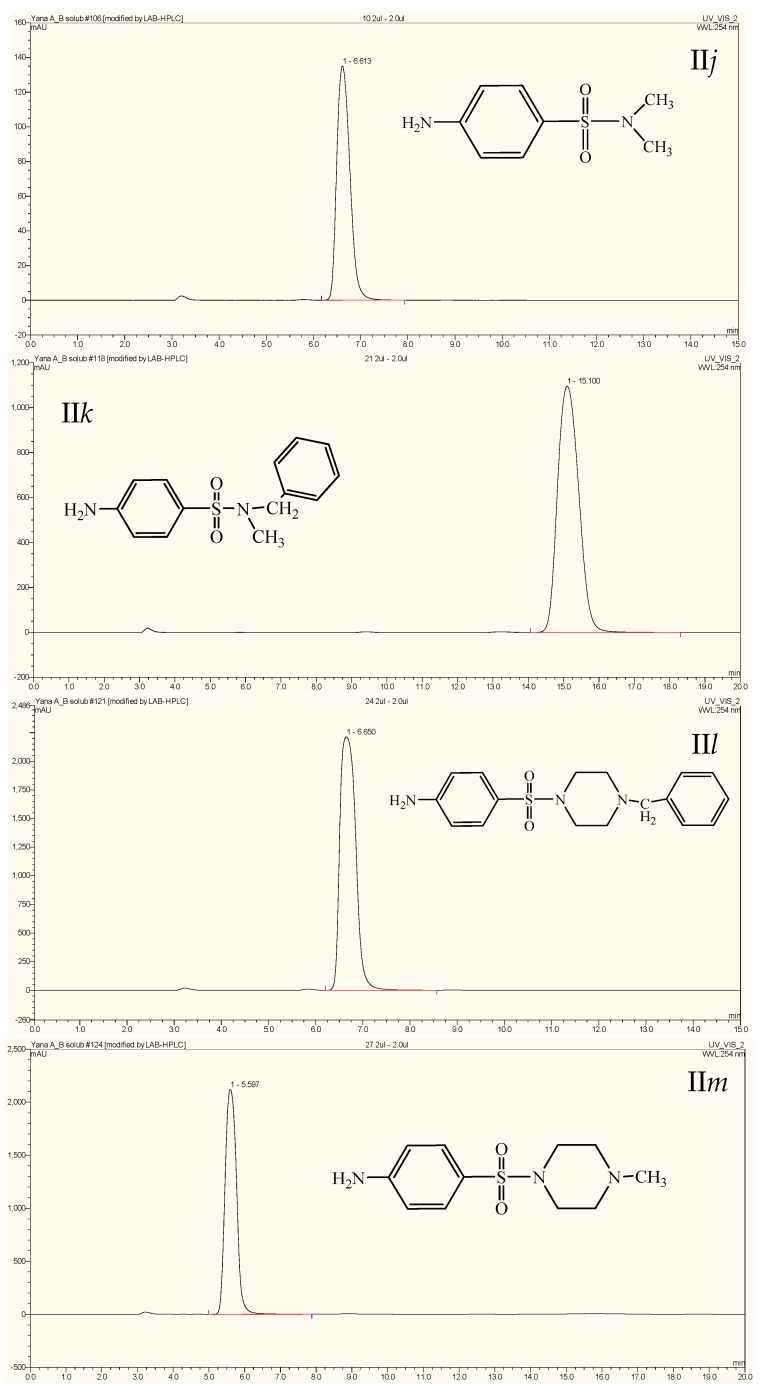

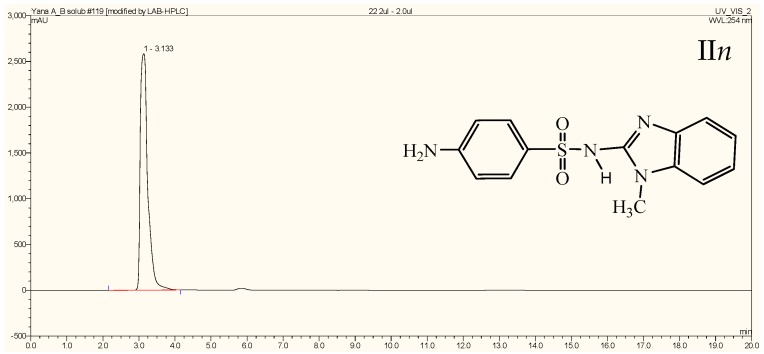
HPLC chromatograms of the synthesized sulfanilamide derivatives **IIa**–***n***.

All compounds were characterized by melting point measurement, by NMR spectra and by HPLC chromatography. Melting points were determined with a Boetius micro melting point apparatus and not corrected. ^1^H NMR spectra were measured with a Brucker HX-270 spectrometer (Bruker Instruments, Billerica, MA, USA) (300 MHz) in acetone-*d*_6_, DMSO-*d*_6_ and CD_3_OD at 298 K. Chemical shifts (δ) are reported in parts per million (ppm) and are referenced to residual signals for deuteriated acetone (δ = 2.07 ppm) and DMSO (δ = 2.50 ppm) and CD_3_OD (δ = 3.34 ppm) as internal standards. Multiplicities are reported as follows: s = singlet, d = doublet, q = quartet, m =multiplet, br = broad, Ar = aromatic, Ph = phenyl, Im = imidazole and coupling constants (J) are given in Hertz. Purity was tested by HPLC analysis using Jasco LC model (Tokyo, Japan) on a RP-18 column YMC-Triart C18, 75 × 3.0 mm, bead size 1.9 μm in the isocratic regime using an eluent composed of 10:40:50 *v*/*v* of 20 mM ammonium acetate: acetonitrile: methanol. Chromatograms of compounds **II*a***–***n*** are presented in Appendix B (Figure A1). Solubility of the compounds **II*a***–***n*** in water was determined in their saturated at ambient temperature aqueous solutions by analyzing saturated aqueous solutions with the help of HPLC using solutions of **II*a***–***n*** with known concentrations in DMSO as standards.

### 4.4. Characterization of Bacterial Strains

Isolation and identification of strains was preformed according to SOPs following standard CLSI procedures. Identification of isolated colonies was performed by the Vitek II system (BioMérieux, Durham, NC, USA) or by matrix-assisted laser desorption ionization–time of flight mass spectrometry (MALDI-TOF MS, BioMérieux). Antibiotic resistance assay was performed in the clinical laboratory by Vitek II system (BioMérieux) using the AST-N270 card and by standard disk diffusion test for *P. aeruginosa* isolates and by standard disk diffusion test for *S. aureus* isolates. Antibiotic resistance/sensitivity tests were performed and interpreted following CLSI guidelines.

### 4.5. Bacterial Cell Growth

Cultures of *S. aureus* and *P. aeruginosa* were grown on brain-heart agar (BHA; Acumedia, Lansing, MI, USA) for 24 h, then transferred into brain-heart broth (BH; Acumedia) and grown overnight at 37 °C and shaking at a 170 rpm. Cell cultures were first diluted by the Antibiotic Medium 3 to obtain an OD_600_ = 0.1, which corresponded to an initial cell concentration of 10^8^ CFU/mL. This concentration was confirmed by a viable cell count [40] in which 100 μL volumes of bacterial samples at various decimal dilutions were evenly spread over BHA plates with a Drigalsky spreader. The plates were incubated at 37 °C overnight and colony forming units (CFU) were counted using a colony counter Scan 500 (Interscience, Saint Nom la Bretèche, France).

### 4.6. PACT Experiments

The minimum inhibitory concentrations (MIC) of Rose Bengal for bacteria were determined by a PACT procedure in transparent glass test tubes as described earlier by us [16]. In brief, Rose Bengal in 2 mL of Antibiotic Medium 3 (hereinafter–medium 3) was distributed by a double dilution method when the Rose Bengal concentration in the first test tube was 40 mg/L. Bacterial suspensions with a concentration of 10^8^ CFU/mL (Section 4.5) were diluted 1:100 with medium 3 and 100 μL of the latter suspensions were then added to 2 mL of medium 3, thus yielding a cell concentration of 5 × 10^4^ CFU/mL. The test tubes, containing bacteria and Rose Bengal, were illuminated for 30 min at ambient temperature by a 18 W white luminescent lamp with an emissions range of 400–700 nm and a light intensity of 1.25 mW/cm^2^ which was measured by a LX-102 Light Meter (Lutron, Taipei, Taiwan). After the illumination, the test tubes were incubated in the dark at 37 °C for 24 h and tested visually for turbidity. MIC values were defined as the lowest concentration of Rose Bengal inhibiting bacterial growth. In all cases, control experiments with bacteria in the presence of Rose Bengal in the dark and in the absence of Rose Bengal under illumination were carried out.

### 4.7. MIC Determination of Sulfanilamide and Its New Derivatives for P. aeruginosa

MIC values of sulfanilamide and its derivatives **II*a***–***n*** were determined by a standard broth double dilutions procedure [41], when the concentration of sulfanilamide and of the derivatives **II*a***–***n*** in the first test tube were correspondent to their solubility in water. The concentration of bacteria in each sample was 5 × 10^4^ CFU/mL. The samples were incubated for 24 h at 37 °C and visually tested for turbidity. The MIC values were defined as the lowest concentration of antimicrobials that inhibited visible growth of bacteria.

### 4.8. MIC Determination of Methicillin for S. aureus

MIC values of methicillin for *S. aureus* were determined in the medium 3 by a standard broth double dilutions procedure [41] using concentration of 19 mg/L in the first test-tube. Concentration of bacteria in all the test-tubes was 5 × 10^4^ CFU/mL. The rest of the procedure was as described in the previous protocol.

### 4.9. Combined PACT-Antibacterial Experiments

Experiments on the combined effect of PACT with methicillin for *S. aureus* and PACT with sulfanilamide for *P. aeruginosa* were performed at sub-MIC concentrations of Rose Bengal which were chosen as half-MIC concentrations for each strain. In each series concentration of Rose Bengal was kept constant and methicillin (in the case of *S. aureus*) or sulfanilamide (in the case of *P. aeruginosa*) were distributed by double dilutions using initial concentrations as described above. Concentration of bacteria in all the test-tubes was 5 × 10^4^ CFU/mL. The rest conditions were as described in the section of PACT experiments. Control experiments on MIC determination in the absence of Rose Bengal, at the sub-MIC concentrations of Rose Bengal without methicillin or sulfanilamide and in the absence of any antimicrobial, were carried out for each series at the conditions of PACT.

### 4.10. SEM Imaging of Bacteria

Suspensions of bacterial cells were centrifugated at 10,000× *g* for 10 min and sediments were treated with 4% paraformaldehyde under gentle mixing for 10 min. The suspensions were centrifugated again and the sediments were washed 6 times by 0.05 M PBS, pH 7.4. The immobilized cells were coated with gold [19]. Micrographs were obtained using a SEM microscope JSM-6510LV (JEOL, Tokyo, Japan).

### 4.11. Reproducibility of the Experiments

In each series, the results were obtained from at least 3 independent experiments with duplicates. Standard errors were calculated for each value. In the case of testing correlation between various factors correlation coefficient R was calculated. The factors were considered independent when R^2^ was less than 0.5.

## 5. Conclusions

Photodynamic inactivation of *S. aureus* and *P. aeruginosa* by Rose Bengal may become an alternative to antibiotic treatment of bacterial infections caused by both antibiotic-sensitive and antibiotic-resistant strains. The antibiotic sensitivity of MRSA strains can be restored by combining methicillin with Rose Bengal. Application of a combined antibiotic-PACT treatment of MRSA cells caused a decrease in the MIC of methicillin. Multi-drug resistant *P. aeruginosa* strains can be eradicated by sulfanilamide. The combination of Rose Bengal and sulfanilamide did not cause any increase in the cells’ sensitivity to sulfanilamide.

## Figures and Tables

**Table 1:** MIC of Rose Bengal, of methicillin alone and in the presence of sub-MIC concentrations of Rose Bengal under illumination for MSSA and MRSA.

Strain of *S. aureus*	MIC of Rose Bengal, mg/L ^1^	MIC of Methicillin, mg/L ^1^	MIC of Methicillin, mg/L ^1^, in the Presence of Sub-MIC Concentration of Rose Bengal	Sub-MIC of Rose Bengal, mg/L ^1^
MSSA 11541	0.625 ± 0.009	1.19 ± 0.02	0.30 ± 0.01	0.313 ± 0.004
MSSA 611971	1.25 ± 0.02	2.38 ± 0.03	0.15 ± 0.01	0.625 ± 0.009
MRSA 69430	2.50 ± 0.03	>19.0 ± 0.3	0.30 ± 0.004	1.25 ± 0.02
MRSA 69397	1.25 ± 0.02	19.0 ± 0.3	<0.0091 ± 0.0002	0.625 ± 0.009
MRSA 69654	1.25 ± 0.02	>19.0 ± 0.3	<0.0091 ± 0.0002	0.625 ± 0.009
MRSA 69740	1.25 ± 0.02	19.0 ± 0.3	0.15 ± 0.01	0.625 ± 0.009
MRSA 69621	1.25 ± 0.02	>19.0 ± 0.3	<0.0091 ± 0.0002	0.625 ± 0.009

^1^ Data are presented as a value ± a standard error.

**Table 2:** MIC values of antibacterial agents for the *P. aeruginosa* antibiotic-sensitive strain 25668 and the antibiotic-resistant strain 63917.

Applied Antimicrobials	MIC, g/L ^1^	
	Strain of *P. aeruginosa*	
	63917	25668
Sulfanilamide	1.75 ± 0.02	3.50 ± 0.08
Rose Bengal	0.78 ± 0.01	0.39 ± 0.01
Sulfanilamide in the presence of Rose Bengal at the sub-MIC concentration	1.75 ± 0.02	3.50 ± 0.08

^1^ Data are presented as a value ± a standard error.

**Table 3:** MIC values of sulfanilamide and its derivatives for the *P. aeruginosa* antibiotic-resistant strain 63917.

Sulfanilamide Derivative	MIC
Sulfanilamide	1.75 ± 0.02 g/L ^1^
**II*a***	>2.68 g/L
**II*b***	>53.7 mg/L
**II*c***	>0.963 g/L
**II*d***	>21.3 mg/L
**II*e***	>8.40 mg/L
**II*f***	>0.102 g/L
**II*g***	>0.157 g/L
**II*h***	>2.74 g/L
**II*i***	>35.7 mg/L
**II*j***	>0.372 g/L
**II*k***	>9.07 mg/L
**II*l***	>26.7 mg/L
**II*m***	>0.447 g/L
**II*n***	>2.10 g/L

^1^ Presented as a value ± a standard error.

## Abbreviations

The following abbreviations are used in this manuscript:

PACT	Photodynamic Antibacterial Chemotherapy
PS	photosensitizer
MSSA	methicillin-sensitive *Staphylococcus aureus*
MRSA	Methycillin-resistant *Staphylococcus aureus*
ROS	reactive oxygen species
MIC	minimum inhibitory concentration
SEM	scanning electron microscope
PBP	penicillin-binding protein
SOP	standard operating procedure
CLSI	The Clinical & Laboratory Standards Institute
CFU	colony forming unit

## Appendix A

### Characteristics of the Synthesized Sulfanilamide Derivatives

*N*-methyl-4-acetylamino benzenesulfonyl amide (**I*a***). Yield 60%; white powder, m.p. 183–185 °C. ^1^H NMR (300 MHz, acetone-*d*_6_): δ = 1.85 (s, 3H, Me), 2.30 (s, 3H, Me), 5.40 (br. s, 1H, NH), 7.40–7.55 (m, 4H, Ar).

*N*-(1-Methylethyl)-4-acetylamino benzenesulfonyl amide (**I*b***). Yield 85.5%, white powder, m.p. 165 °C. ^1^H NMR (300 MHz, CD_3_OD): δ = 1.10 (s, 9H, 3 Me), 2.10 (s, 3H, Me), 7.50 (d, *J* = 6.5 Hz, 2H, Ar), 7.60 (d, *J* = 6.5 Hz, 2H, Ar).

*N*-(1,1-Dimethylethyl)-4-acetylamino benzenesulfonyl amide (**I*c***), Yield 91%, white powder, m.p. 205 °C. ^1^H NMR (300 MHz, CD_3_OD): δ = 1.10 (s, 9H, 3 Me), 2.10 (s, 3H, Me), 7.50 (d, *J* = 6.5 Hz, 2H, Ar), 7.60 (d, *J* = 6.5 Hz, 2H, Ar).

*N*-Phenyl-4-acetylamino benzenesulfonyl amide (**I*d***), Yield 67.2%, white powder, m.p. 207–209 °C. ^1^H NMR (300 MHz, acetone-*d*_6_): δ = 1.85 (s, 3H, Me), 6.80–7.30 (m, 9H, Ar), 7.43 (br. s, 1H, NH), 8.6 (br. s, 1H, NH).

*N*-(2-Methoxyphenyl)-4-acetylamino benzenesulfonyl amide (**I*e***), Yield 71.8%, white powder, m.p. 161 °C. ^1^H NMR (300 MHz, acetone-*d*_6_): δ = 2.10 (s, 3H, Me), 2.60 (s, 3H, Me), 3.80 (br. s, 1H, NH), 7.00–7.90 (m, 8H, Ar).

*N*-Benzyl-4-acetylamino benzenesulfonyl amide (**I*f***), Yield 89.3%, white powder, m.p. 158 °C. ^1^H NMR (300 MHz, acetone-*d*_6_): δ = 2.10 (s, 3H, Me), 4.35–4.42 (m, 2H, CH_2_), 4.80 (br. s, 1H, NH), 7.00–7.90 (m, 9H, Ar).

*N*-(3-Fluorobenzyl)-4-acetylamino benzenesulfonyl amide (**I*g***). Yield 86%, white powder, m.p. 139 °C. ^1^H NMR (300 MHz, DMSO-*d*_6_): δ = 2.09 (s, 3H, Me), 3.99 (s, 2H, CH_2_), 7.01–7.08 (m, 2H, Ar), 7.08 (d, *J* = 7.72 Hz, 1H, Ar), 7.25–7.33 (m, 1H, Ar), 7.70–7.75 (m, 4H, Ar), 8.07 (s, 1H, NH), 10.29 (s, 1H, NH).

*N*-(Carboxypropyl)-4-acetylamino benzenesulfonyl amide (**I*h***), Yield 75%, white powder, m.p. 145 °C. ^1^H NMR (300 MHz, DMSO-*d*_6_): δ = 1.57 (d, *J* = 7.23 Hz, 2H, CH_2_), 2.10 (s, 3H, Me), 2.19 (t, *J* = 7.36 Hz, 2H, CH_2_), 2.66 (q, *J* = 6.72 Hz, 2H, CH_2_), 5.50 (s, 1H, NH), 7.02–7.06 (m, 2H, Ar), 7.25–7.35 (m, 1H, Ar), 11.2 (s, 1H, NH).

*N*,*N*-Diphenyl-4-acetylamino benzenesulfonyl amide (**I*i***), Yield 91.6%, white powder, m.p. 233 °C. ^1^H NMR (300 MHz, acetone-*d*_6_): δ = 2.05–2.14 (m, 3H, Me), 2.90 (s, 1H, CH), 5.70 (br. s, 1H, NH), 7.20–07.6 (m, 14H, Ar).

*N*,*N*-Dimethyl-4-acetylamino benzenesulfonyl amide (**I*j***), Yield 80%, white powder, m.p. 149 °C. ^1^H NMR (300 MHz, acetone-*d*_6_): δ = 2.20 (s, 3H, Me), 2.70 (s, 6H, 2 Me), 5.40 (br. s, 1H, NH), 6.60–7.50 (m, 4H, Ar).

*N*-Benzyl-*N*-methyl-4-acetylamino benzenesulfonyl amide (**I*k***), Yield 78%, white powder, m.p. 154 °C. ^1^H NMR (300 MHz, acetone-*d*_6_): δ = 2.20 (s, 3H, Me), 2.60 (s, 3H, MeCO), 4.20 (s, 2H, CH_2_), 7.25–7.36 (m, 5H, Ph), 7.70–7.90 (m, 4H, Ar).

(4-Benzylpiperazino)-4-acetylamino benzenesulfonyl amide (**I*l***), Yield 69%, white powder, m.p. 108 °C. ^1^H NMR (300 MHz, acetone-*d*_6_): δ = 2.10 (s, 3H, Me), 2.40–2.55 (m, 4H, 2 CH_2_), 2.90–3.00 (m, 4H, 2 CH_2_), 3.45–3.55 (m, 2H, CH_2_-Ph), 7.10–7.25 (m, 5H, Ph), 7.60–7.80 (d, *J* = 6.5 Hz, 4H, Ar), 9.50 (br. s, 1H, NH).

(4-Methylpiperazino)-4-acetylamino benzenesulfonyl amide (**I*m***), Yield 81%, white powder, m.p. 198 °C. ^1^H NMR (300 MHz, acetone-*d*_6_): δ = 2.05 (s, 3H, Me), 2.13 (s, 3H, Me), 2.33 (s, 4H, 2 CH_2_), 2.78 (s, 4H, 2 CH_2_), 7.60 (d, *J* = 6.5 Hz, 2H, Ar), 7.70 (d, *J* = 6.5 Hz, 2H, Ar), 10.5 (br. s, 1H, NH).

*N*-(1-Methylbenzimidazole-2-yl)-4-acetylamino benzenesulfonyl amide (**I*n***). Yield 24%, white powder, m.p. 168 °C. ^1^H NMR (300 MHz, DMSO-*d*_6_): δ = 2.05–2.15 (m, 3H, Me), 3.20 (s, 3H, Me), 4.70 (br. s, 1H, NH), 7.10–7.80 (m, 8H, Ar), 10.70 (br. s, 1H, NH).

Sulfanilic acid *N*-methyl amide (**II*a***), Yield 48%; m.p. 110 °C. ^1^H NMR (300 MHz, acetone-*d*_6_): δ = 1.85 (s, 3H, Me), 5.00 (br. s, 2H, NH_2_), 6.60 (d, *J* = 6.5 Hz, 2H, Ar), 7.50 (d, *J* = 6.5 Hz, 2H, Ar).

Sulfanilic acid *N*-(1-methylethyl) amide (**II*b***). Yield 68%, white powder, m.p. 116 °C. ^1^H NMR (300 MHz, acetone-*d*_6_): δ = 1.04 (s, 3H, Me), 1.06 (s, 3H, Me), 3.25–3.35 (m, 1H, CH), 6.75 (d, *J* = 6.5 Hz, 2H, Ar), 7.55 (d, *J* = 6.5 Hz, 2H, Ar).

Sulfanilic acid *N*-(1,1-dimethylethyl) amide (**II*c***). Yield 87%, white powder, m.p. 126 °C. ^1^H NMR (300 MHz, acetone-*d*_6_): δ = 1.20 (s, 9H, 3 Me), 5.30 (br. s, 2H, NH_2_), 5.90 (br. s, 1H, NH), 6.70 (d, *J* = 6.5 Hz, 2H, Ar), 7.70 (d, *J* = 6.5 Hz, 2H, Ar).

Sulfanilic acid *N*-phenyl amide (**II*d***). Yield 93%, white powder, m.p. 197 °C. ^1^H NMR (300 MHz, acetone-*d*_6_): δ = 5.40 (br. s, 2H, NH_2_), 6.60–6.90 (m, 4H, Ar), 7.00–7.60 (m, 5H, Ar), 8.20 (br. s, 1H, NH).

Sulfanilic acid *N*-(2-methoxyphenyl) amide (**II*e***). Yield 97.2%, white powder, m.p. 205 °C. ^1^H NMR (300 MHz, DMSO-*d*_6_): δ = 3.70 (s, 3H, Me), 5.40 (br. s, 2H, NH_2_), 6.65 (d, *J* = 6.5 Hz, 2H, Ar), 6.90 (d, *J* = 6.5 Hz, 2H, Ar), 7.05–7.15 (m, 1H, Ar), 7.46–7.50 (m, 3H, Ar), 7.64 (br. s, 1H, NH).

Sulfanilic acid *N*-benzyl amide (**II*f***). Yield 99%, white powder, m.p. 120 °C. ^1^H NMR (300 MHz, DMSO-*d*_6_): δ = 4.60 (s, 2H, CH_2_), 5.40 (br. s, 2H, NH_2_), 6.65–6.75 (m, 7H, Ar), 6.90 (d, *J* = 6.5 Hz, 2H, Ar).

Sulfanilic acid *N*-(3-fluorophenyl) amide (**II*g***). Yield 97%,white powder, m.p. 120 °C. ^1^H NMR (300 MHz, DMSO-*d*_6_): δ = 3.91 (d, *J* = 1.5 Hz, 2H, CH_2_), 5.90 (s, 1H, Ar), 6.60 (d, *J* = 8.63 Hz, 1H, Ar), 7.00–7.06 (m, 2H, Ar), 7.08 (d, *J* = 7.69 Hz, 1H, Ar), 7.32 (dd, *J* = 7.35 and 14.33 Hz, 1H, Ar), 7.43 (d, *J* = 8.61 Hz, 2H, Ar), 7.69 (t, *J* = 2.25 Hz, 1H, NH).

Sulfanilic acid *N*-(carboxypropyl) amide (**II*h***). Yield 86%, white powder, m.p. 129 °C. ^1^H NMR (300 MHz, DMSO-*d*_6_): δ = 1.57 (q, *J* = 7.23 Hz, 2H, CH_2_), 2.19 (t, *J* = 7.36 Hz, 2H, CH_2_), 2.66 (q, *J* = 7.23 Hz, 2H, CH_2_), 5.88 (br. s, 2H, NH_2_), 6.60 (d, *J* = 8.63 Hz, 2H, Ar), 7.01 (t, *J* = 5.92 Hz, 1H, NH), 7.39 (d, *J* = 8.62 Hz, 2H, Ar), 11.99 (br. s, 1H, OH).

Sulfanilic acid *N*-(bis-phenylmethyl) amide (**II*i***). Yield 97%, white powder, m.p. 179 °C. ^1^H NMR (300 MHz, DMSO-*d*_6_): δ = 2.90 (s, 1H, CH), 5.30 (br. s, 1H, NH), 5.60 (br. s, 2H, NH_2_), 6.60 (d, *J* = 6.5 Hz, 2H, Ar), 7.15–7.30 (m, 10H, Ar), 7.60 (d, *J* = 6.5 Hz, 2H, Ar).

Sulfanilic acid *N*-(1,1-dimethyl) amide (**II*j***). Yield 94%, white powder, m.p. 175 °C. ^1^H NMR (300 MHz, acetone *d*_6_): δ = 2.50 (s, 6H, 2 Me), 5.40 (br. s, 2H, NH_2_), 6.60–7.50 (m, 4H, Ar).

Sulfanilic acid *N*-methyl-*N*-benzyl amide (**II*k***). Yield 98%, white powder, m.p. 137 °C. ^1^H NMR (300 MHz, acetone-*d*_6_): δ = 2.50 (s, 3H, Me), 4.20 (s, 2H, CH_2_), 5.50 (br. s, 2H, NH_2_), 6.70 (d, *J* = 6.5 Hz, 2H, Ar), 7.25–7.35 (m, 5H, Ph), 7.60 (d, *J* = 6.5 Hz, 2H, Ar).

Sulfanilic acid *N*-(4-benzylpiperazino) amide (**II*l***). Yield 89%, white powder, m.p. 244 °C. ^1^H NMR (300 MHz, acetone-*d*_66_ + DMSO-*d*_6_ (1:1 *v*/*v*)): δ = 2.35–2.45 (m, 4H, (CH_2_)_2_N), 2.40–2.50 (m, 4H, (CH_2_)_2_N-SO_2_), 3.30 (br. s, 2H, NH_2_), 3.40 (s, 2H, CH_2_-Ph), 6.60–7.40 (m, 4H, Ar), 7.30–7.40 (m, 5H, Ph).

Sulfanilic acid *N*-(4-methylpiperazino) amide (**II*m***). Yield 92%, white powder, m.p. 212 °C. ^1^H NMR (300 MHz, DMSO *d*_6_): δ = 2.13 (s, 3H, Me), 2.33 (s, 4H, (CH_2_)_2_N), 2.78 (s, 4H, (CH_2_)_2_N-SO_2_), 6.10 (br. s, 2H, NH_2_), 6.65 (d, *J* = 8.45 Hz, 2H, Ar), 7.34 (d, *J* = 8.36 Hz, 2H, Ar).

Sulfanilic acid *N*-(1-methylbenzimidazole-2-yl) amide (**II*n***). Yield 45%, white powder, m.p. 265 °C. ^1^H NMR (300 MHz, acetone-*d*_6_ + DMSO-*d*_6_ (1:1 *v*/*v*)): δ = 3.40 (s, 3H, Me), 5.60 (br. s, 2H, NH_2_), 6.60 (d, *J* = 8.1 Hz, 2H, Ar), 7.50 (d, *J* = 8.2 Hz, 2H, Ar), 7.10–7.80 (m, 4H, Ar_im_).
